# Geomorphological characteristics of the Wabash River, USA: Influence on fish assemblages

**DOI:** 10.1002/ece3.7349

**Published:** 2021-03-18

**Authors:** Jeff Robbins, Mark Pyron

**Affiliations:** ^1^ Department of Biology Ball State University Muncie IN USA

**Keywords:** fish assemblage, functional process zones, functional traits, river geomorphology

## Abstract

River hydrogeomorphology is a potential predictor of ecosystem and assemblage variation. We tested for fish assemblage variation as a function of hydrogeomorphology in a Midwestern US large river, the Wabash River. Fish data were classified by taxonomy and traits and we tested if assemblages varied with river hydrogeomorphology or river distance, defined into 10‐km distinct reaches. Three unique geomorphological units, Functional Process Zones (FPZ), were identified using an ArcGIS hydrogeomorphic model, based primarily on channel width, floodplain width, and down valley slope. Five locations were identified as FPZ A with narrow stream channel, high down valley slope, and an expansive floodplain. Ten locations were identified as FPZ B with a wide river channel and wide floodplain. Thirty‐five locations were identified as FPZ C with wide river channel and a constrained floodplain. The sites were categorized into three stream orders: 5, 6, and 7. We found hydrogeomorphology classified by unique FPZs or by river distance influenced taxonomic and functional fish assemblages for the Wabash River. There was high overlap among fish occurrences among FPZs, but nine species resulted as significant indicators of specific FPZs. Five traits were significant indicators of FPZs: an intermediate Swim Factor score, medium tolerance to silt, small‐large stream size preference, and two Shape Factor categories. Our conclusions are that fish assemblages respond strongly to local geomorphology and river distance, fitting the riverine ecosystem synthesis and the river continuum concept.

## Graphical abstract

We tested for fish assemblage variation as a function of hydrogeomorphology in a Midwestern US large river, the Wabash River. Fish data were classified by taxonomy and traits and we tested if assemblages varied with river hydrogeomorphology or river distance, defined into 10‐km distinct reaches. Our conclusions are that fish assemblages respond strongly to local geomorphology and river distance, fitting the riverine ecosystem synthesis and the river continuum concept.

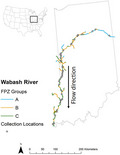

## 1 INTRODUCTION

Riverine fish assemblages vary due to evolutionary history, past river connections (Wiley & Mayden, 1985), and habitat variation (Lyons, 1996; Matthews, 1998). Studies of habitat variation and fish assemblage responses are frequently at local assemblage scales (Matthews et al., 1994), although additional spatial scales of catchment, reach, and sites can be included (Gido et al., 2006). The habitat that fishes utilize is primarily a result of river geomorphology interacting with hydrology (Delong et al., 2019; Walters et al., 2003). Fish assemblages can be successfully linked to instream geomorphological attributes of fish habitats (Lamouroux et al., 2002; Walters et al., 2003). For example, Delong et al. (2019) found that interactions of hydrology and geomorphic heterogeneity defined local habitats and acted as an environmental filter for fish assemblages.

Stream ecosystem attributes can be used to validate discrete changes in stream morphology and instream habitat. Thorp et al. (2006) and Maasri et al. (2019) demonstrated that stream assemblages and ecosystem processes are structured by hydrogeomorphology. Macroinvertebrate assemblages vary with hydrogeomorphology in tropical streams (Godoy et al., 2016) and temperate streams (Collins et al. (2018). Food chain length varies among functional process zones (FPZ) defined by geomorphology (Thoms et al., 2017). Maasri et al. (Submitted) found strong patterns for geomorphological variation with beta diversity of Mongolia stream fishes.

Stream fishes can be classified by habitat and trophic preferences or traits that include water column position, diet, and river size (Matthews, 1998; Poff & Allan, 1995). Fishes defined by traits for habitat use, life history strategy, locomotion, and feeding varied predictably with longitudinal stream morphology in high elevation rivers (Pease et al., 2012). Functional trait analyses of fish assemblages result in more detailed recognition of river ecosystem variation. Fishes classified by trophic guild, stream size preference, water movement preference, and other characteristics link organisms to ecosystems. This trait or functional approach was the basis for previous community ecology comparisons to standardize biologic integrity evaluations (Karr et al., 1986).

Fishes have distinctive preferences for habitat features including water velocity, substrate type, and instream cover (Angermeier & Karr, 1984; Angermeier & Schlosser, 1989). These habitat variables are predicted to vary with geomorphology and with river distance, resulting in fish assemblage variation. We tested if the geomorphology of a river defined by geomorphology using a RESonate model (Williams et al., 2013) predicts fish assemblage variation defined by taxonomy and by functional traits. This model uses digital elevation models, precipitation, geology, and downloadable tools (www.macrorivers.org) to categorize river patches with a self‐emerging statistical procedure, without prior classification (Maasri et al., [Link], 2019, Submitted). Analyzing fish assemblage variation by geomorphology will further validate the RESonate model. In addition, we tested if river location explains fish assemblage variation. Our primary objective was to categorize the Wabash River mainstem using geomorphology data and test if taxonomic and functional fish assemblages vary among geomorphologically distinct reaches or with river location. We further tested if species or functional traits were significant indicators for FPZs or stream order, and we tested for spatial autocorrelation among fish species and trait abundances with river location (Grennouillet et al., 2008).

## 2 METHODS

### 2.1 Site locations

**FIGURE 1:**
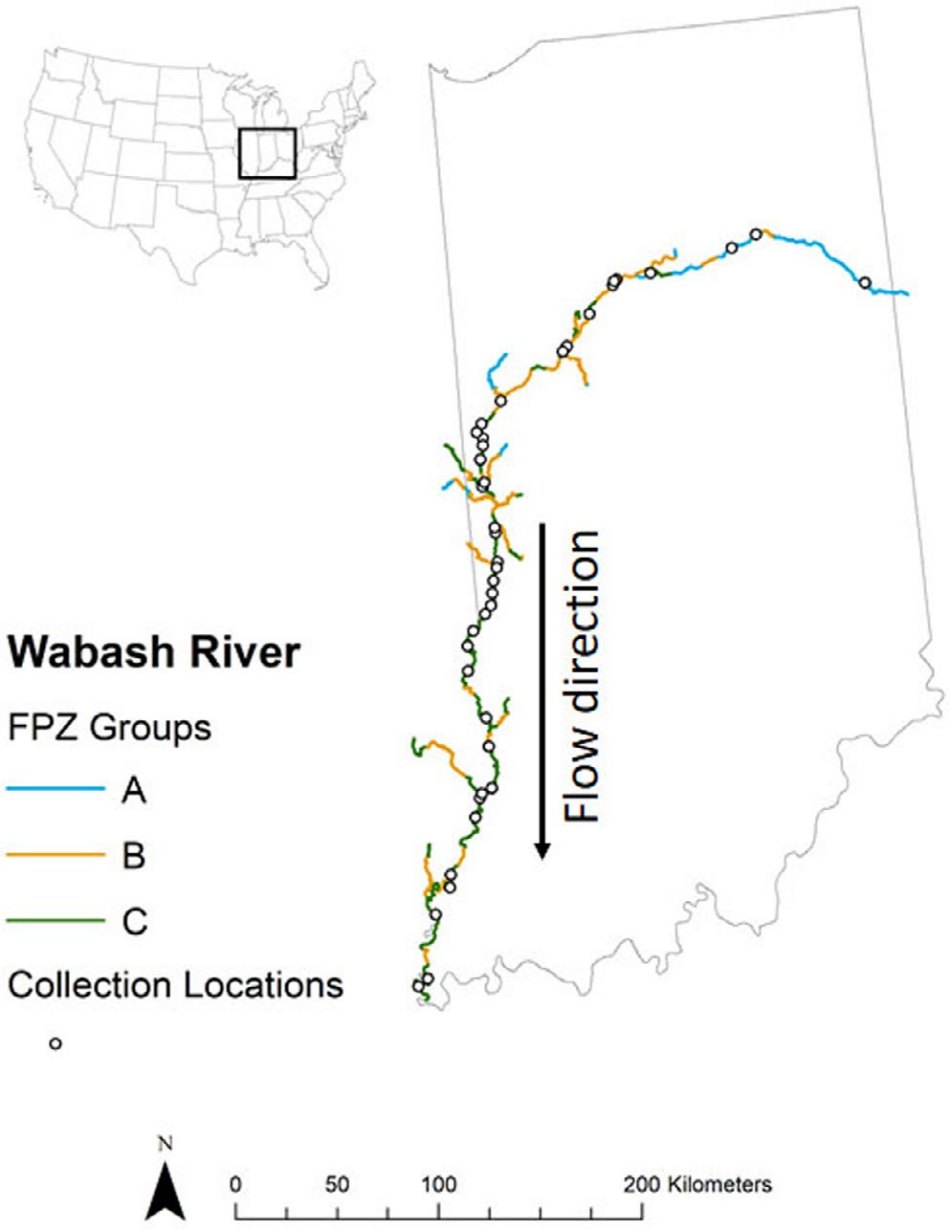
The Wabash River with repeated functional process zones A, B, and C generated from RESonate and locations for fish collections

We studied the Wabash River (Figure 1) and we used the ArcGIS hydrogeomorphic model RESonate to identify distinctive reaches or FPZs (Kotlinski et al., 2016) following Williams et al. (2013) and Maasri et al. (Submitted). We extracted these variables at 10 km intervals: elevation, mean annual precipitation, geology, valley width, valley floor (floodplain) width, valley width‐to‐valley floor width ratio, river channel sinuosity, right valley slope, left valley slope, and down valley slope. Data were normalized to a 0 to 1 scale and a dissimilarity matrix was generated using a Gower dissimilarity transformation (Gower, 1971). The Gower transformation is recommended for nonbiological data when the measures are range‐standardized (Thoms et al., 2018). The dissimilarity matrix was used in a hierarchical clustering following the Ward linkage method, as it provided the best partitioning of cluster groups (Murtagh & Legendre, 2014). Additionally, we used a Principal Component Analysis (PCA) to identify the contributive variables most important for group partitioning, and to describe the cluster groups based on the ten variables identified above. Groups were later mapped to allow for the identification of collection sites. We performed the clustering using the *cluster* package (version 2.1.0) (Maechler et al., 2018) and the PCA using the *FactoMineR* package (version 1.42) (Lê et al., 2008) in R version 3.6.3 (R Core Team, 2020). We mapped the resulting groups using ArcGIS (version 10.5).

We further defined river locations using the Strahler stream order method to delineate on a longitudinal scale (Strahler, 1957). We used the hydrology tools in the Spatial Analyst toolbox in ArcGIS to determine stream orders for our sites. Stream order calculation followed methods of Tarboton et al. (1991) using digital elevation models.

The main channel of the Wabash River was analyzed from the headwaters near Celina, Ohio to the confluence with the Ohio River, a 764‐km distance (Gammon, 1998). The watershed area of the Wabash River is 85,340 km^2^ (Benke & Cushing, 2005) and has a single impoundment at Huntington, Indiana at rkm 662 but multiple other dams are on tributaries. The Wabash River valley has relatively low topographical variation throughout the longitudinal river gradient (Gammon, 1998). The mainstem Wabash River has predominantly natural sinuosity and relatively high variation in aquatic habitats due to limited human alterations of the channel form (Pyron & Lauer, 2004). Multiple extenuated bends occur near the Ohio River confluence where the river gradient is low. However, multiple hydrologic alterations are present for the Wabash River from tributary reservoir release patterns and rowcrop agricultural water management (Pyron & Neumann, 2008).

### 2.2 Fish collections

Fish data for the Wabash River were obtained from the Indiana Department of Environmental Management (IDEM). We used 20 years of fish collections conducted by IDEM via boat and tow barge electrofishing for 500‐m reaches on the mainstem Wabash River. IDEM collection sites were randomized and included only upstream locations to the Wabash River–Ohio River confluence. We used data for 32 Wabash River sites by including more than a single collection date for several sites that were sampled more than once. Our data were fish species and abundances at each site.

### 2.3 Data analyses

We followed Poff and Allan (1995) for trait categories of trophic guild, stream size preference, current velocity preference, substratum preference, tolerance to silt, and body morphology as used by Pyron et al. (2011). Trophic guilds included were general invertivores, omnivores, and benthic invertivores; body morphology was determined by swim factor and shape factor (Appendix S1).

Fish species relative abundance data (by site using CPUE for 500‐m reaches) were analyzed after arcsine square root transformation to reduce the impact of high abundances at several sites. All species were included in analyses. We used nonmetric multidimensional scaling (NMDS) with Bray–Curtis dissimilarity to visualize occurrence data by functional process zones and by river distance. NMDS axes were examined for significant correlations with river distance and with stream order. MANOVAs (Minitab 18, minitab.com) were used to test if taxonomic and functional fish assemblage variation by FPZs or river distance were significant. We used CANOCO 5 (canoco5.com) for NMDS, to test for indicator species (and trait categories) by FPZ and stream order using 999 permutations to test significance, and for Mantel tests using Bray–Curtis distances and 999 permutations to test significance. We were interested if particular species or trait categories occurred more than expected with river distance, and if fish assemblages exhibit spatial autocorrelation. Mantel tests test for concordance between two distance‐based variables (Grennouillet et al., 2008).

## 3 RESULTS

The RESonate model resulted in three unique FPZs for the Wabash River (Figure 1). FPZs A, B, and C were repeated longitudinally throughout the mainstem of the river with A zones predominantly upstream, and C zones downstream with B zones interspersed throughout the river length. Five locations were diagnosed as FPZ A, with a narrow valley width, narrow valley floor width with little to no down valley slope (gradient). Ten locations were diagnosed as FPZ B, with a wide river channel paired with the widest floodplain values and a moderate current. There were 35 locations diagnosed as FPZ C, with a wide river channel, but the most constrained floodplain of all three FPZs (Table 1). The IDEM Wabash River fish data included 99 fish species from 50 sites (Appendix S2). Functional process zones A contained 58 species, B zones contained 70 species, and C zones contained 82 species. Our results for defining stream order from ArcGIS software resulted in stream orders 5, 6, and 7 for the Wabash River. Stream order 5 contained 2 sites (3 collections) and 36 total species collected. There were 9 sites (16 collections) in stream order 6 that resulted in 84 total species. A total of 21 sites (31 collections) in stream order 7 that resulted in 75 total species.

**TABLE 1:** Mean geomorphological variables (±*SD*) for the Wabash River based upon 10‐km stream reaches

	Elevation (m)	Valley width (m)	Valley floor width (m)	Ratio of VW to VFW	Left valley slope	Right valley slope	Down valley slope	River Sinuosity
FPZ A	215 (25)	4,945 (2,664)	114 (185)	88 (55)	0.04 (0.08)	0.002 (0.05)	0.00052 (0.0003)	1.4 (0.22)
FPZ B	152 (28)	8,667 (1796)	214 (495)	150 (135)	0.01 (0.01)	0.013 (0.06)	0.00012 (0.0002)	1.5 (0.22)
FPZ C	132 (18)	3,528 (2,443)	205 (138)	21 (13)	0.03 (0.04)	0.021 (0.03)	0.00012 (0.0001)	1.5 (0.54)

**FIGURE 2:**
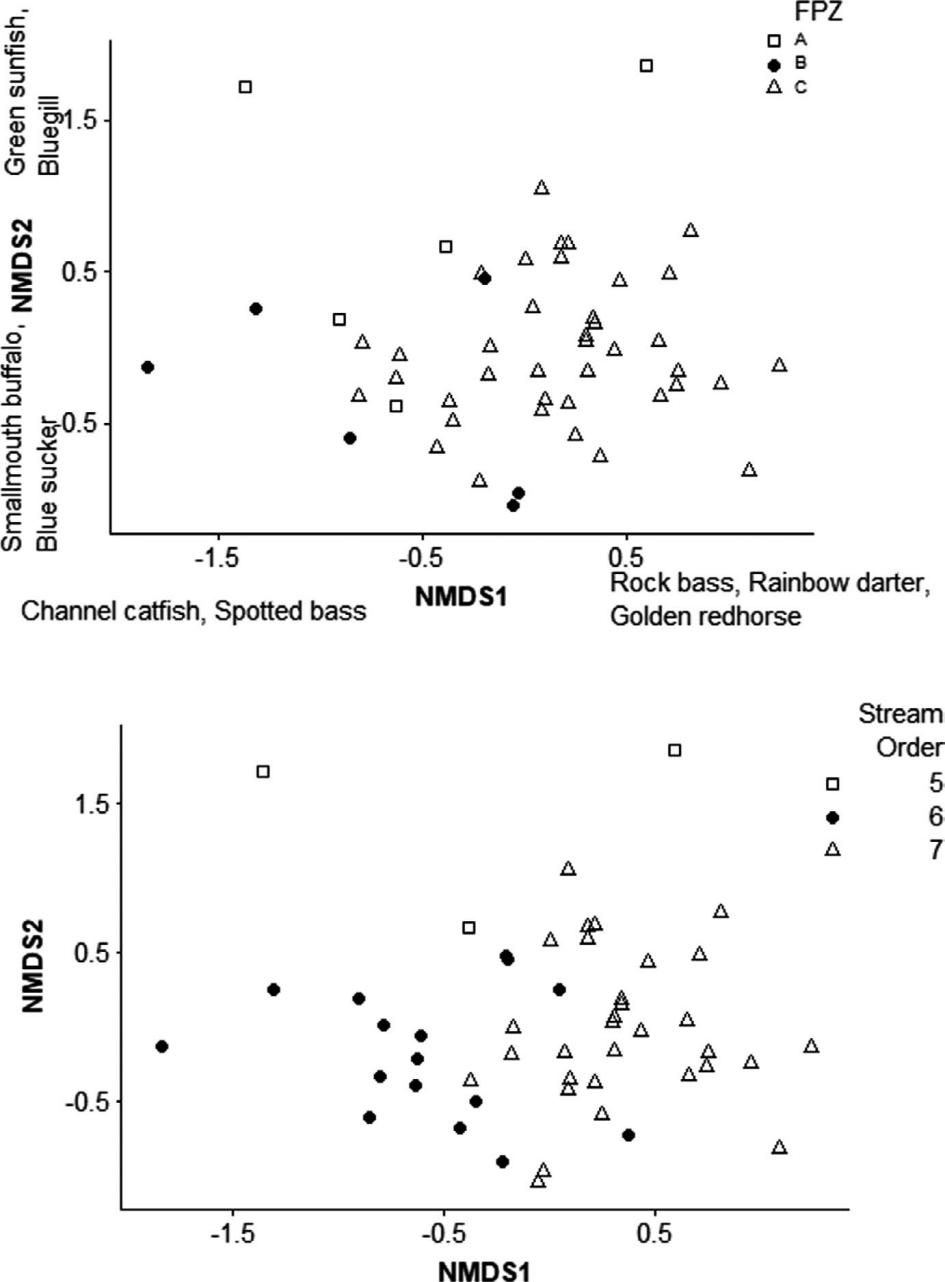
NMDS ordinations for Wabash River fish species presented by FPZs (stress = 0.15, top) and by stream order (bottom). FPZs are A, B, and C. Stream orders are 5, 6, and 7. Highest species loadings are indicated on axes

The NMDS using taxonomy resulted in a stress value of 0.15 and two axes that explained 41% and 36.3% of variation (Figure 2). The first NMDS axis was significantly correlated with river distance (*r* = .71, *p* < .001). The first and second NMDS axes were significantly correlated with stream order (NMDS1 *r* = .61, *p* < .001; NMDS2 *r* = −0.3, *p* = .03). Taxonomic structure of fish assemblages differed among the three FPZs (MANOVA Wilks’ *F*_4,92_ = 5.9, *p* < .001; Figure 2). In addition, taxonomic structure of fish assemblages differed by stream order (MANOVA Wilks’ *F*_4,92_ = 16.8, *p* < .001).

**FIGURE 3:**
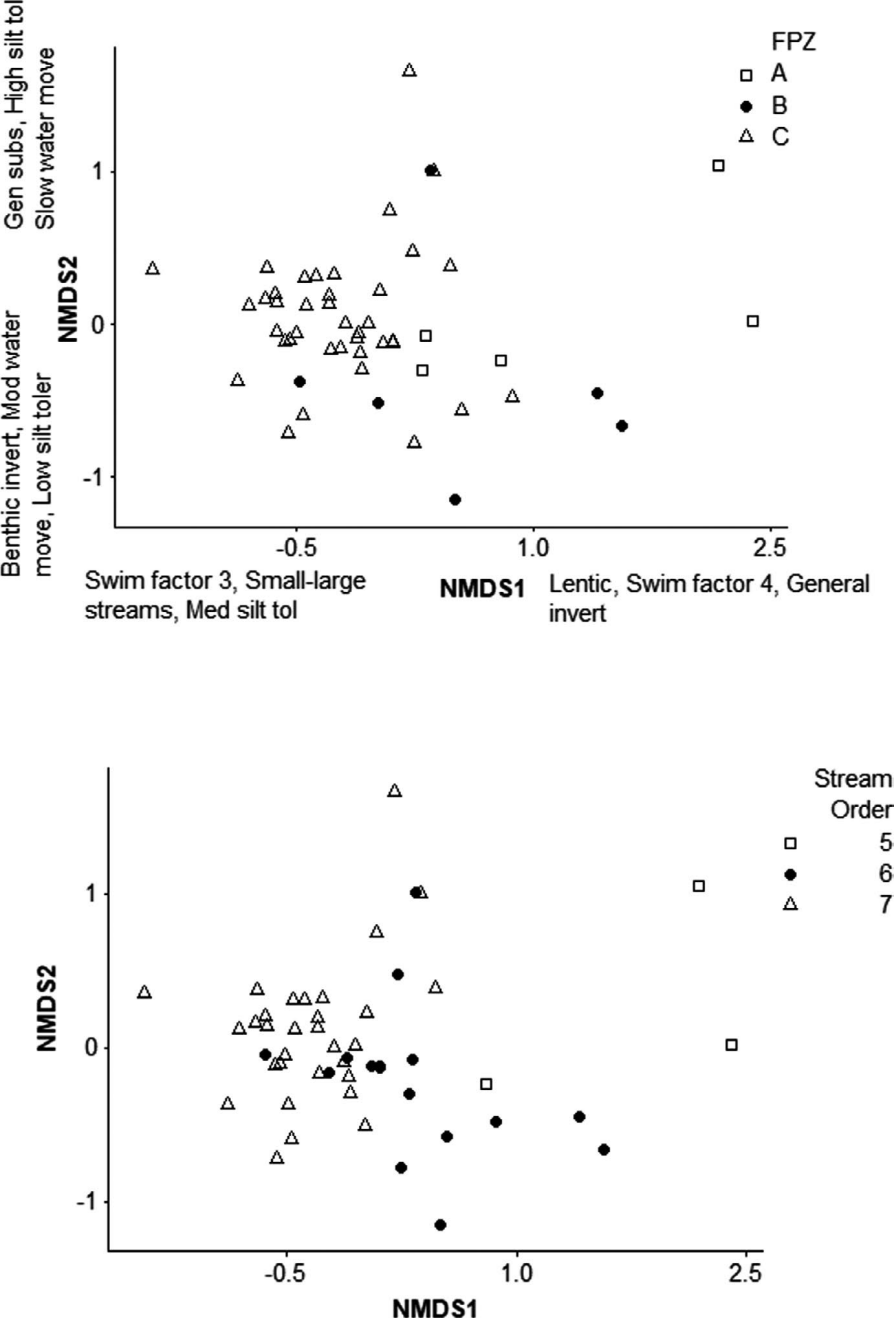
NMDS ordinations for Wabash River fish functional traits presented by FPZs (stress = 0.1, top) and by stream order (bottom). FPZs are A, B, and C. Stream orders are 5, 6, and 7. Highest species loadings are indicated on axes

Relative abundances of fishes classified into traits varied by FPZ. The NMDS using functional traits resulted in a stress value of 0.1 and two axes that explained 52.1% and 25.2% of variation (Figure 3). Both functional trait NMDS axes were significantly correlated with river distance (NMDS1 *r* = −.7, *p* < .001; NMDS2 *r* = .3, *p* < .04). NMDS1 was significantly correlated with stream order (*r* = −.74, *p* < .001). Fish assemblages classified into functional traits also differed between three FPZs in the Wabash River (MANOVA Wilks’ *F*_4,56_ = 6.1, *p* < .001; Figure 3). In addition, functional traits of fish assemblages differed by stream order (MANOVA Wilks’ *F*_4,92_ = 15.1, *p* < .001).

There was high overlap among fishes among FPZs but nine species resulted as significant indicators of FPZs (Table 2). Species that were indicators for FPZ A were tadpole madtom (*Noturus gyrinus* Mitchill, 1817), central stoneroller (*Campostoma anomalum* Rafinesque, 1820), and river redhorse (*Moxostoma carinatum* Cope, 1870). Species that were indicators for FPZ B were mountain madtom (*Noturus eleutherus* Jordan, 1877), golden redhorse (*Moxostoma erythrurum* Rafinesque, 1818), and river shiner (*Notropis blennius* Girard, 1856). Species that were indicators for FPZ C were spotted bass (*Micropterus punctulatus* Rafenisque, 1819), freshwater drum (*Aplodinotus grunniens* Rafinesque, 1819), and emerald shiner (*Notropis atherinoides* Rafinesque, 1818). Five traits were significant indicators of FPZs (Table 2, Appendix S2): Shape Factor 2.5–3.5 was an indicator for FPZ A, Shape Factor 12.5–13.5 was an indicator for FPZ B. An intermediate Swim Factor score, medium tolerance to silt, and small‐large stream size preference were indicators for FPZ C.

**TABLE 2:** Significant indicator species analysis results for the Wabash River. Analyses were for species and for traits as indicators for FPZs (top) and stream order (lower)

Species	A	B	C	Preferred FPZ	*p*
Wabash river species					
Mountain madtom	0.00	0.67	0.00	B	.008
Spotted bass	0.18	0.00	0.66	C	.009
Golden redhorse	0.10	0.71	0.02	B	.012
River shiner	0.00	0.78	0.10	B	.016
Tadpole madtom	0.50	0.00	0.00	A	.021
Freshwater drum	0.25	0.08	0.61	C	.027
Central stoneroller	0.47	0.00	0.00	A	.041
Emerald shiner	0.01	0.13	0.67	C	.041
River redhorse	0.47	0.00	0.00	A	.041
Wabash River Traits					
Swim Factor 0.35–0.45	0.22	0.33	0.46	C	.002
Medium Tolerance to Silt	0.19	0.34	0.47	C	.005
Small‐Large Stream Size	0.26	0.29	0.45	C	.015
Shape Factor 2.5–3.5	0.51	0.20	0.29	A	.019
Shape Factor 12.5–13.5	0.06	0.66	0.04	B	.025

Species	5	6	7	Preferred stream order	*p*
Wabash river species					
Black redhorse	0.00	0.50	0.00	6	.008
Bullhead minnow	0.06	0.47	0.20	6	.007
Channel catfish	0.05	0.39	0.44	7	.002
Emerald shiner	0.06	0.20	0.49	7	.002
Golden redhorse	0.08	0.57	0.01	6	.045
Longear sunfish	0.18	0.42	0.23	6	.031
Orangespotted sunfish	0.48	0.02	0.02	5	.044
River carpsucker	0.00	0.54	0.38	6	.001
Sauger	0.09	0.45	0.02	6	.037
Steelcolor shiner	0.00	0.44	0.04	6	.020
Tadpole madtom	0.61	0.01	0.00	5	.007
Wabash River Traits					
Herbivore–detritivore	0.01	0.66	0.30	6	.038
Moderate Water Movement	0.19	0.34	0.47	6	.012
General Water Movement	0.13	0.61	0.25	6	.006
Swim Factor 0.45–0.55	0.64	0.30	0.06	5	.040
Shape Factor 1.5–2.5	0.71	0.24	0.04	5	.009

There was high overlap among fishes among stream orders and 11 species resulted as significant indicators of stream orders (Table 2). Species that were indicators for stream order 5 were orangespotted sunfish (*Lepomis humilis* Girard, 1858) and tadpole madtom. Species that were indicators for stream order 6 were black redhorse (*Moxostoma duquesnii* Lesueur, 1817), bullhead minnow (*Pimephales vigilax* Baird and Girard, 1853), golden redhorse, longear sunfish (*Lepomis megalotis* Rafinesque, 1820), river carpsucker (*Carpiodes carpio* Rafinesque, 1820), sauger (*Sander canadensis* Griffith and Smith, 1834), and steelcolor shiner (*Cyprinella whipplei* Girard, 1856). Species that were indicators for stream order 7 were channel catfish (*Ictalurus punctatus* Rafinesque, 1818), and emerald shiner. Five traits were significant indicators of stream order (Table 2): Swim Factor 0.45–0.55 and Shape Factor 1.5–2.5 were indicators for stream order 5. Herbivore–detritivore trophic traits, moderate water movement, and general water movement preferences were indicators for stream order 6.

The Mantel test for taxonomic fish assemblages with river distance was significant (Mantel *r* = .51, *p* = .001). The Mantel test using functional traits with river distance was significant (Mantel *r* = .19, *p* = .036). These results indicate the presence of spatial autocorrelation for fish assemblages.

## 4 DISCUSSION

We found distinct patterns for fish assemblages among three unique FPZs and by stream order in the Wabash River. The three FPZs we identified differ primarily based on floodplain width (valley width and valley floor width) and river gradient (valley slope). These three geomorphological variables resulted in distinctive habitat variation among FPZs such that fish assemblages varied among FPZs. Thorp et al. (2008) and Wolter et al. (2016) predicted distinct ecosystem functioning and assemblage variation among FPZs with different geomorphology as tenets of the river ecosystem synthesis. Our results for fish assemblage variation with river distance also support the river continuum concept (Vannote et al., 1980).

Stream size preferences of fishes (Beugly & Pyron, 2010) among Wabash River FPZ’s appear to contribute to the distinctive fish assemblage structure we detected. Fishes that prefer small‐ and medium‐sized streams including yellow bullhead, tadpole madtom, and orangethroat darter were collected in FPZ A with narrow channels and a wide floodplain that result in slow water movement. Species that prefer wide channels and wide floodplains that result in moderate current velocity were Tippecanoe darter, streamline chub, black redhorse, and logperch were collected in FPZ B. FPZ C had wide channels and a constrained floodplain and fish assemblages in these FPZs included increased white bass, Mississippi silvery minnow (*Hybognathus nuchalis*, Agassiz, 1855), and black buffalo (*Ictiobus niger* Rafinesque 1819). Trophic differences for fishes explained variation in the abundances of fishes among FPZs. For example, surface and water column invertivores like emerald shiner, silver shiner (*Notropis photogenis* Cope, 1865), and spotfin shiner (*Cyprinella spiloptera* Cope, 1867) were collected in FPZ C, similar to Broadway et al. (2015) and Poff and Allan (1995). Functional Process Zone A consisted of a narrow channel width with lentic structure. Species in FPZ A tended to have low silt tolerance and swim factors for streamlining. Functional Process Zone C had a large channel width resulting in moderate to fast current that is suitable for fishes with streamlined swim factor. This suggests that fishes in larger and higher current velocity locations of the Wabash River require increased hydrodynamic shape to inhabit those environments (Langerhans, 2008; Webb & Weihs, 1986). These results fit predictions of the river ecosystem synthesis model where unique geomorphological river reaches contain unique fish assemblage structure (Thorp et al., 2021, 2008). Our results for fish assemblage variation with river distance and stream order also fit the river continuum concept (Vannote et al., 1980). Other river ecosystem models may also fit our results (Thorp et al., 2021).

The data we used were from 20 years of collections with temporal variation that we were unable to incorporate into analyses. We predict that this additional source of variation contributed to a lack of stronger results for some analyses. We recommend future studies test for temporal variation in fish assemblages that were collected at the same locations. Delineating FPZs and associating them with the ecology of a river can help predict locations of endangered and management species. Determining the presence of economical and conservational valued fishes could promote better management decisions based on FPZ type, and conservation and restoration projects to better protect species of concern.

## CONFLICT OF INTEREST

There are no sources or any potential sources of conflict of interest. There is no interest or relationship, financial or otherwise, that might be perceived as influencing the objectivity of this.

## AUTHOR CONTRIBUTIONS

**Jeff Robbins:** Conceptualization (equal); Methodology (equal); Visualization (equal); Writing‐original draft (equal). **Mark Pyron:** Conceptualization (supporting); Resources (lead); Writing‐review & editing (equal).

## ETHICAL APPROVAL

Fish data were obtained from a government agency, not collected by authors.

## Supporting information

Appendix S1

Appendix S2

## Data Availability

Data are available as appendices.
