# Supplementary material for: Geomorphological characteristics of the Wabash River, USA: Influence on fish assemblages
Source: Ecol Evol. 2021 Mar 18;11(9):4542–9. doi: 10.1002/ece3.7349 (PMC8093669; doi:10.1002/ece3.7349)
Supplement: Supplementary file 1 — Appendix S1 [file ECE3-11-4542-s002.docx]

**Appendix 1** Functional traits from Poff and Allan (1995**)**

| **Trophic Guild**  TRO1 Herbivore-detritivore  TRO2 Omnivore  TRO3 General Invertivore  TRO4 Surface/Water Column **Invertivore**  TRO5 Benthic Invertivore  TRO6 Piscivore  TRO7 Planktivore  TRO8 Parasite  **Stream Size Preference**  SS1 Small  SS2 Medium-Large  SS3 Small-Large  SS4 Lentic  **Current Velocity Preference**  WM1 Fast  WM2 Moderate  WM3 Slow-None  WM4 General  **Substratum Preference**  SUB1 Rubble (rocky, gravel)  SUB2 Sand  SUB3 Silt  SUB4 General  **Tolerance To Siltation**  TOL1 High  TOL2 Medium  TOL3 Low  **Swim Factor**  SwF1 0.15-0.25  SwF2 0.25-0.35  SwF3 0.35-0.45  SwF4 0.45-0.55  SwF5 0.55-0.65  SwF6 0.65-0.75  SwF7 0.75-0.85  SwF8 0.85-0.95  **Shape Factor**  ShF1 0.0-1.5  ShF2 1.5-2.5  ShF3 2.5-3.5  ShF4 3.5-4.5  ShF5 4.5-5.5  ShF6 5.5-6.5  ShF7 6.5-7.5  ShF8 7.5-8.5  ShF9 8.5-9.5  ShF10 9.5-10.5  ShF11 10.5-11.5  ShF12 11.5-12.5  ShF13 12.5-13.5  ShF14 13.5-14.5 |
| --- |
